# The Central Nervous System and Psychosocial Factors in Primary Microvascular Angina

**DOI:** 10.3389/fcvm.2022.896042

**Published:** 2022-05-13

**Authors:** Mattia Cattaneo, Geza Halasz, Magdalena Maria Cattaneo, Adel Younes, Camilla Gallino, Isabella Sudano, Augusto Gallino

**Affiliations:** ^1^Cardiology Department, Istituto Cardiocentro Ticino, Lugano, Switzerland; ^2^Human Medicine Department, Università della Svizzera italiana, Lugano, Switzerland; ^3^Cardiovascular Research Unit, Hospital of San Giovanni, Bellinzona, Switzerland; ^4^Heart Failure Unit, Guglielmo da Saliceto Hospital, Azienda unità sanitaria locale (AUSL) Piacenza, University of Parma, Parma, Italy; ^5^Human Medicine Department, University of Zurich, Zurich, Switzerland; ^6^Cardiology Department, University Hospital, University Heart Center Zurich, Zurich, Switzerland

**Keywords:** primary microvascular angina, central nervous system, psychosocial factors, unconventional interventions, spinal cord stimulation, pain modulation centers

## Abstract

Patients diagnosed with ischemia without obstructive coronary artery disease (INOCA) comprise the group of patients with primary microvascular angina (MVA). The pathophysiology underlying ischemia and angina is multifaceted. Differences in vascular tone, collateralization, environmental and psychosocial factors, pain thresholds, and cardiac innervation seem to contribute to clinical manifestations. There is evidence suggesting potential interactions between the clinical manifestations of MVA and non-cardiac conditions such as abnormal function of the central autonomic network (CAN) in the central nervous system (CNS), pain modulation pathways, and psychological, psychiatric, and social conditions. A few unconventional non-pharmacological and pharmacological techniques targeting these psychosocial conditions and modulating the CNS pathways have been proposed to improve symptoms and quality of life. Most of these unconventional approaches have shown encouraging results. However, these results are overall characterized by low levels of evidence both in observational studies and interventional trials. Awareness of the importance of microvascular dysfunction and MVA is gradually growing in the scientific community. Nonetheless, therapeutic success remains frustratingly low in clinical practice so far. This should promote basic and clinical research in this relevant cardiovascular field investigating, both pharmacological and non-pharmacological interventions. Standardization of definitions, clear pathophysiological-directed inclusion criteria, crossover design, adequate sample size, and mid-term follow-up through multicenter randomized trials are mandatory for future study in this field.

## Key Points

- Evidence suggests a potential role of the central nervous system and psychosocial conditions in the clinical manifestations of microvascular angina.
- A few unconventional non-pharmacological approaches have shown preliminary and encouraging results.
- The results of the available observational and interventional trials have multiple drawbacks and a low level of evidence.
- Standardization of definitions, pathophysiological-directed inclusion criteria, adequate sample size, and mid-term follow-up are mandatory for future research.

## Introduction

### Rationale

For decades, physicians have considered obstructive coronary artery disease (CAD) as an equivalent to ischemia and angina. However, the pathophysiology underlying ischemia and angina is more complex and multifaceted. Anatomical and functional alterations of both epicardial vessels and microcirculation, left ventricular mass and fibrosis, and non-cardiac components contribute to the clinical manifestations.

Large prospective registries such as the Women's Ischemic Syndrome Evaluation (WISE) database and other seminal studies showed that angina in the absence of obstructive CAD affects up to 60% of patients undergoing diagnostic coronary angiography for the assessment of stable angina, a larger number of patients than previously thought (1, 2). Subjects diagnosed with ischemia without obstructive coronary artery disease (INOCA) include the group of patients with primary microvascular angina (MVA), formerly known as coronary syndrome X. MVA is defined as the clinical manifestation of myocardial ischemia caused by coronary microvascular dysfunction (CMD) in the absence of obstructive CAD, epicardial coronary vasospasm, and structural heart disease (3, 4).

**Figure 1:**
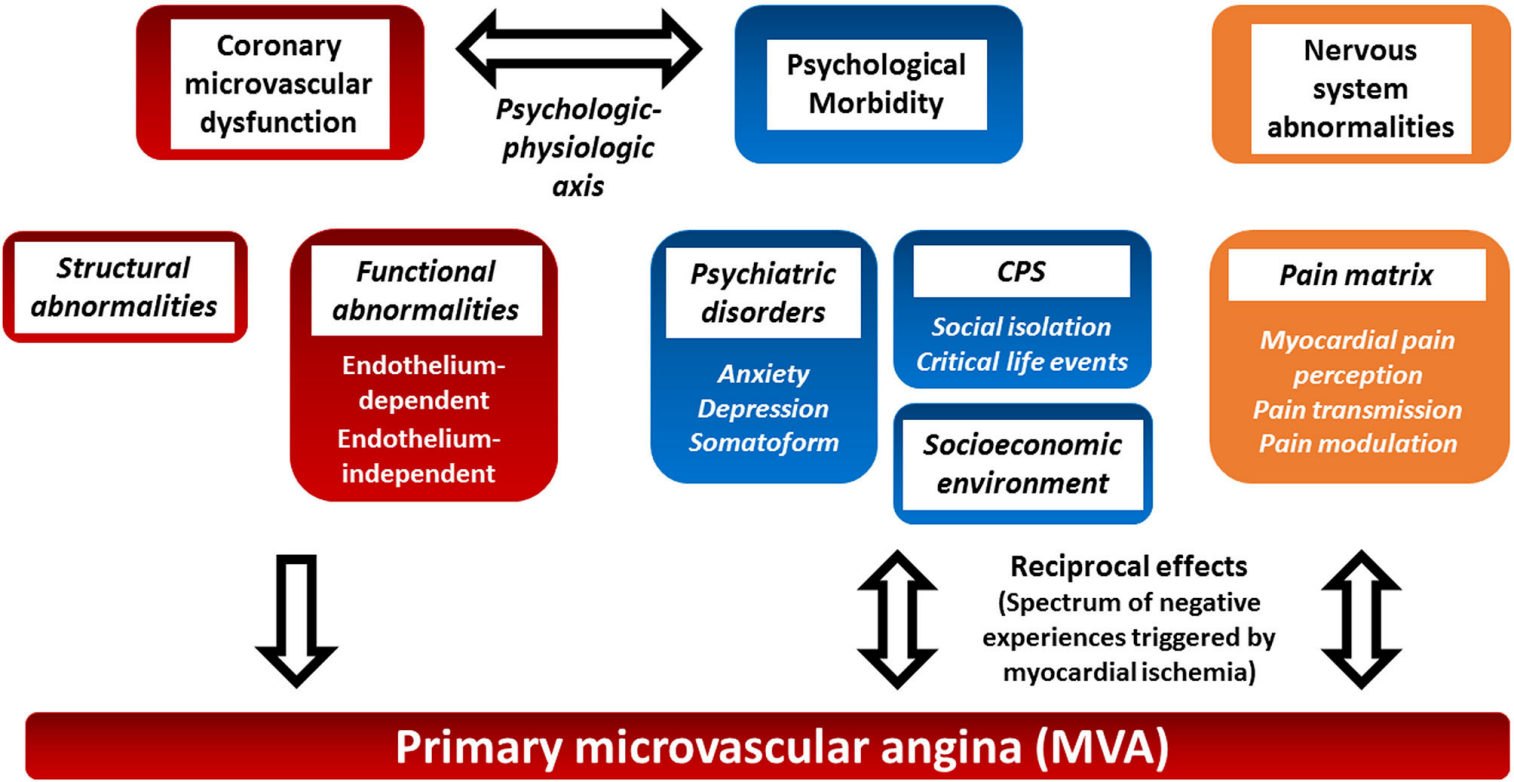
Proposed pathophysiology of primary microvascular angina and potential role of psychosocial factors and the central nervous system. Primary microvascular angina (MVA), formerly known as coronary syndrome X, is defined as the clinical manifestation of myocardial ischemia caused by coronary microvascular dysfunction (CMD) in the absence of obstructive CAD, epicardial vasospasm, and structural heart disease. Structural and functional coronary microcirculation alteration results in CMD. The clinical manifestation of MVA, psychosocial factors, and the central nervous system autonomic/afferent pathways may have reciprocal multifaceted activities (see text for details). CMD, coronary microvascular dysfunction; CPS, chronic psychosocial stress; MVA, primary microvascular angina.

Coronary microvascular dysfunction can result from a combination of structural and functional alterations of the coronary microcirculation (5). The relative importance of structural and functional changes varies in different clinical settings (Figure 1) (5). Structural abnormalities result in progressive reduction in the microcirculation bed that mimics the effects of flow limiting obstructive CAD. Functional abnormalities include endothelium-dependent, endothelial-independent vasodilatation impairments, and excessive microvascular constriction (5).

However, the pathophysiology of MVA is even more complex. Actually, the relationship between ischemia and angina is not straightforward in both CAD and microvascular dysfunction (6). The so-called ischemic threshold demonstrates intraindividual and interindividual variability (7, 8). Differences in vascular tone, collateralization, environmental and psychosocial factors, pain thresholds, and cardiac innervation seem to play a role in such variability (7–9). The ORBITA trial has stressed the benefits of the placebo effect and the role of psychosocial factors in stable macrovascular CAD (10, 11). This review focuses on the role of the central nervous system, chronic psychological stress, and psychosocial stressors in the settings of MVA (Figure 1). The evidence on the role of psychosocial factors in MVA is limited, but they have been suggested to play a possible relevant role in the clinical manifestations of the latter. Moreover, a few non-pharmacological interventions have been attempted to control the symptoms.

Since those concepts have been recently introduced in cardiovascular medicine, we also provided a brief overview of the most relevant pathophysiological and clinical literature for each topic of the review to introduce the reader.

### Objectives and Study Selection

The objectives of the review were to examine and gather evidence from observational and interventional studies on the role of the central nervous system and psychosocial factors in primary microvascular angina, formerly known as cardiac syndrome X.

## Discussion

### Atypical Manifestations and Female Preponderance in MVA

Atypical clinical manifestations are more common in patients with MVA than in those with obstructive CAD (12). Moreover, it is recognized that age and gender influence variations in the clinical manifestation of MVA (4). This suggests that non-cardiac factors such as hormonal production, neurological pain pathway modulation, and concurrent psychosocial conditions may all contribute to the pathogenesis as well as the clinical manifestations of MVA (12).

In most of the reported studies, there is a robust female preponderance among subjects with MVA. The large multicenter WISE study showed that nearly 50–70% of women and 30–50% of men were diagnosed with INOCA, respectively (13). A few non-randomized studies characterized by small sample numbers have even stronger female preponderances ranging from 50 to 100%.

### Psychosocial Factors in MVA

#### Chronic Psychosocial Stress

Chronic psychosocial stress (CPS) is a fundamental component of life, but its definition is complex. Many definitions of stress exist, making the study of the impact of CPS on cardiovascular disease challenging. CPS disrupts both health and wellbeing. These are multidimensional, intertwined entities that include both objective and subjective elements, as well as physical, mental, social, and economic dimensions (14). CPS can arise from multiple sources such as psychiatric disorders, major life challenges, critical life-events, poor-quality relationships, social isolation, ethnic discrimination, job-strain, and adverse socioeconomic factors (15, 16). Large prospective cohort studies and meta-analysis corroborate the existing link between CPS and cardiovascular diseases (CVD). The magnitude of risk for major cardiovascular events (MACEs) associated with CPS may be equal to that attributed to the traditional CVD risk factors (16, 17). These risk ratios for chronic psychosocial stress are estimated mostly between 1.2 and 2.0 (15, 16). These ratios depend on both the population and the cardiovascular outcome being studied. The most consistent evidence is from psychiatric disorders such as depression and anxiety disorders that have been associated with an increased risk of incident coronary heart disease (CHD), hypertension, myocardial infarction, and stroke (15). Estimates for associations of negative psychological factors with cardiovascular disease are more consistent for incident CHD, incident cardiovascular events, and stroke (15). Interestingly, in a study from the Swedish National Patient Register with stress-related disorders, overall stress-related disorders were strongly associated with early onset cardiovascular diseases (hazard ratio 1.40), and this association was not modified by the presence of psychiatric comorbidity, except for fatal cardiovascular events (18).

A further complication to measure the magnitude of association between CPS, the above-mentioned heterogeneous stressors, and CVD is the fact that chronic stress also promotes CVD risk factors. Moreover, as classical cardiovascular risk factors, these stressors tend to cluster (15, 16). This clustering challenges the appreciation of the relative risk of each individual stressor and CPS itself, as well as the definitions of causal relationships. Moreover, the interactions between the level of stressor exposure and each individual's physiological response to a given stressor exposure, namely, the perceived stress, determine effects on health (15, 16). Perceived stress has been associated with an increased (RR, 1.27) risk of incident CHD and CHD mortality regardless of the stressor (19).

Last but not least, an increasing wealth of data shows that psychological health can positively or negatively affect cardiovascular health, but cardiovascular diseases can mutually affect the former (20).

Mental stress-induced myocardial ischemia (MSIMI) is a plain manifestation linking CPS with CVD. It has been associated with future MACEs (21). Notably, both MVA and MSIMI, as well as mental stress-induced endothelial dysfunction, have a meaningfully higher prevalence in women than in men compared to age-matched men (5, 22, 23). No specific risk ratios exist for the association of MVA and CPS unrelated to the cause of the latter.

#### Mind-Heart-Body Connection

**Figure 2:**
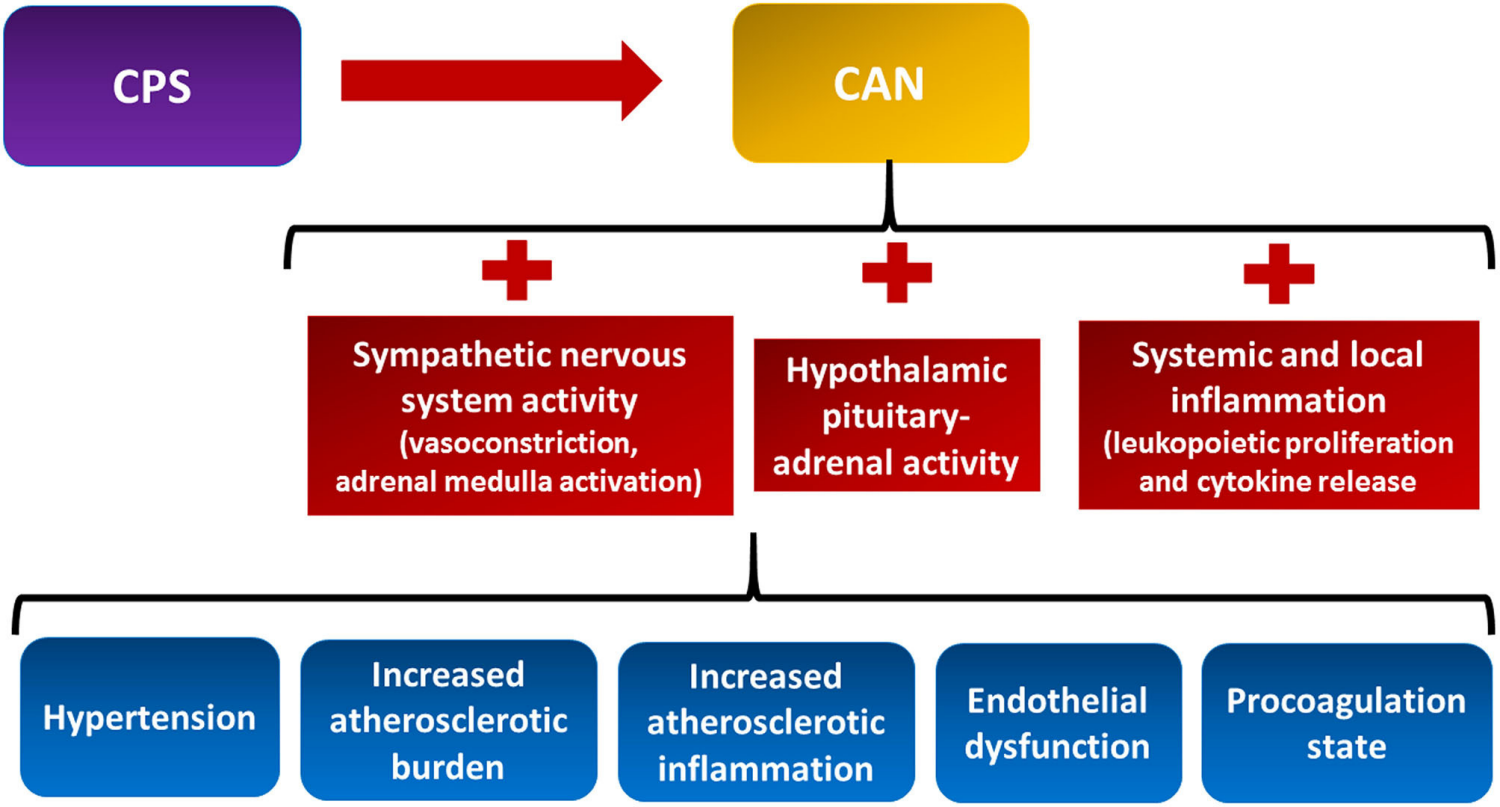
The neuro–immune–arterial axis. It is the multisystem pathophysiological processes linking chronic psychological stress (CPS) and cardiovascular disease. CPS triggers dysregulation of the autonomic nervous system, the hypothalamic pituitary–adrenal axis, the local and systemic inflammation, and the coagulation system (see text for details). CAN, central autonomic network.

The intertwined relationship between the heart, body, and mind has been defined as the mind-heart-body (MHB) connection (20). Within the MHB connection, the psychological–physiological axis, namely, the pathophysiological processes linking CPS and CVD have only recently begun to be clarified. Convincing evidence exists that psychosocial stress perturbs the central nervous system (24). The areas of the CNS that modulate the autonomic response to external stressors are collectively called the central autonomic network (CAN) (25) (Figure 2, yellow box). The CAN comprises the limbic system that plays a pivotal function to actuate the response to both acute and chronic psychological stressors (25). CPS perturbs the activities of the CAN that result in dysregulation of the autonomic nervous system, the hypothalamic–pituitary–adrenal (HPA) axis, local and systemic inflammation, and the coagulation system (26) (Figure 2, red boxes). This neuro–immune–arterial axis causes accelerated atherosclerosis, increased atherosclerotic burden, and plaque inflammation, as well as endothelial dysfunction with altered vascular reactivity and procoagulation state (Figure 2, blue boxes) (20, 27, 28).

Stress activates the HPA axis. This results in the release of pituitary adrenocorticotropic hormone (ACTH), which induces the adrenal cortex to produce glucocorticoids that contribute to increased adiposity, hypertension, and insulin resistance (29). Sympathetic hyperactivity induces vasoconstriction and increases peripheral vascular resistance and heart rate, thus promoting higher blood pressure and lower heart rate variability, both related to adverse cardiovascular events. Moreover, sympathetic activation of the adrenal medulla results in the systemic release of catecholamines that multiplies the sympathetic response (30). CPS also promotes immune system dysregulation. This perturbation accounts for transcription of pro-inflammatory genes, bone marrow leukopoietic proliferation, pro-inflammatory cytokine release, and leukocyte infiltration of the intima (31–33). While evidence of the association between MVA, CPS, and many stressors exists, much has to be clarified regarding the role of the neuro–immune–arterial axis in MVA.

#### Chronic Psychological Stress in MVA

Several methods can be used to measure stress. Psychometric questionnaires are the most widely implemented tools in this research field, but recently, functional imaging of the central nervous system has gained interest (27, 34).

No specific measurements of the overall level of perceived stress as well as specific functional cerebral imaging of CPS in MVA are present in the literature. Most studies in MVA investigated quality of life (QoL) as well as the distinct components of the psychosocial dimension that can result in CPS.

#### Psychiatric Comorbidities

Psychological and psychiatric risk factors seem to be a higher burden for women with INOCA and MVA than for men (23). As mentioned above, evidence of an increased risk of incident CHD and stroke in subjects affected by depression and anxiety disorders is consistent. Among psychiatric disorders, depression and anxiety have been shown to play a role in MVA and INOCA in the large WISE database (35–37).

Smaller cross-sectional studies tried to investigate the prevalence of anxiety and depression in MVA by implementing largely validated questionnaires such as the Hospital Anxiety and Depression Scale, Beck Anxiety Inventory (BAI), Beck Depression Inventory (BDI), and Health-Related Quality of Life (HRQoL) (36-Item Short Form Health Survey). The results of these studies suggest that patients with MVA have a higher prevalence of CPS, psychiatric disorders, and lower quality of life (38–40). Anxiety disorders (up to 64% prevalence), depression, and somatoform disorders represented the three most prevalent disorders, respectively. The clinical utility of depression measures in combination with measures of anxiety has been confirmed by Rutledge et al. who screened 489 women using the BDI and State-Trait Anxiety Inventory (STAI), respectively. Participants were followed for a median of 5.9 years, showing that the value of depression for predicting CVD events varied by the severity of concurrent anxiety (41). Additionally, the pilot study by Vermeltfoort et al. indicated that subjects with MVA affected by the highest trait anxiety assessed by the STAI showed the largest area of ischemia assessed by myocardial perfusion scintigraphy (42). Moreover, heart-focused health anxiety, namely the anxiety related to potential and actual cardiovascular diseases and symptoms, has been shown to occur more frequently in women than in men (43). Furthermore, in two small observational studies, about 30% of patients with MVA met the diagnostic criteria of third edition of *Diagnostic and Statistical Manual of Mental Disorders* (*DSM*) for panic disorder (44, 45).

Interestingly, anxiety has also been associated with a conspicuous increased risk of epicardial coronary artery spasm (RR, 5.20; 95% CI: 4.72–5.40) (46), but the risk associated with anxiety for increased microvascular spasm is not currently known in MVA.

Altogether, these results showed an association between psychiatric disorder and angina in MVA. However, their relative importance in the pathogenesis of MVA and the reciprocal influence between psychiatric comorbidities and clinical manifestation of MVA remain to be further elucidated (Figure 1).

#### Social and Educational Factors

Psychosocial stress is highly correlated with socioeconomic, behavioral, dietary, and environmental risk factors as well as the decreased access to healthcare system (16). Gender differences in socioeconomic conditions have been shown in numerous large observational registries, including most of the cardiovascular diseases. Women with INOCA are more likely to belong to ethnic minorities, have a lower income, and have a history of abuse and harassment (16, 23). The study by Asbury et al. (47) showed that women with MVA suffered higher levels of critical life events and had smaller social network in comparison to patients with obstructive CAD and controls.

#### Symptoms and Quality of Life Assessment

Angina pain triggered by ischemia is frequently associated with dyspnea, fatigue, discomfort, and possibly fear. Pain represents a major concern for HRQoL in individuals with MVA. Patients with MVA showed poorer scores on various HRQoL scales compared to both patients with obstructive CAD and normal control (39, 48). MVA is associated with impaired quality of life, higher risk of disability, greater physical limitation, and higher angina prevalence compared to either matched stable obstructive CAD population or acute myocardial infarction population (39, 49–52). We recently reported a small analysis of the SF-36 questionnaire showing a discrepancy between the higher level of self-reported body pain and the higher mental health score in MVA as compared to patients with CAD and Takotsubo cardiomyopathy. This may suggest a mechanism of somatization since patients affected by different somatization phenomena report considerable physical impairment as compared to the general population, independently from the slightly reduced mental health score (53). Consistently, somatoform disorders are one of the most prevalent DSM diagnoses in patients with INOCA and MVA (39, 54).

Patients with MVA often experience refractory angina, leading to repeated outpatient visits, hospitalizations, and unnecessary and potentially harmful diagnostic procedures that may contribute to poor HRQoL (55). In the original WISE cohort during 5-year follow-up, patients with INOCA had elevated rates of hospital readmission (four times higher than men within 180 days) and repeat angiography and had an increased risk rate for MACEs of at least 2.5% (56).

#### Work Limitations and Economy

Individuals diagnosed with MVA have been reported to have frequent absences from work and work limitations (49).

The substantial economic burden and healthcare expenditures in patients with INOCA and MVA are similar to those in patients with obstructive CAD in the United States and Europe (47, 57).

### Pain and Angina-Like Symptoms Perception and Modulation

#### Transmission and Modulation of Cardiac Pain

Pain represents a single component of the large spectrum of the subject's negative experiences triggered by myocardial ischemia. Dyspnea, fatigue, dyspepsia, discomfort fear, and a sense of imminent death may rarely convoy angina.

**Figure 3:**
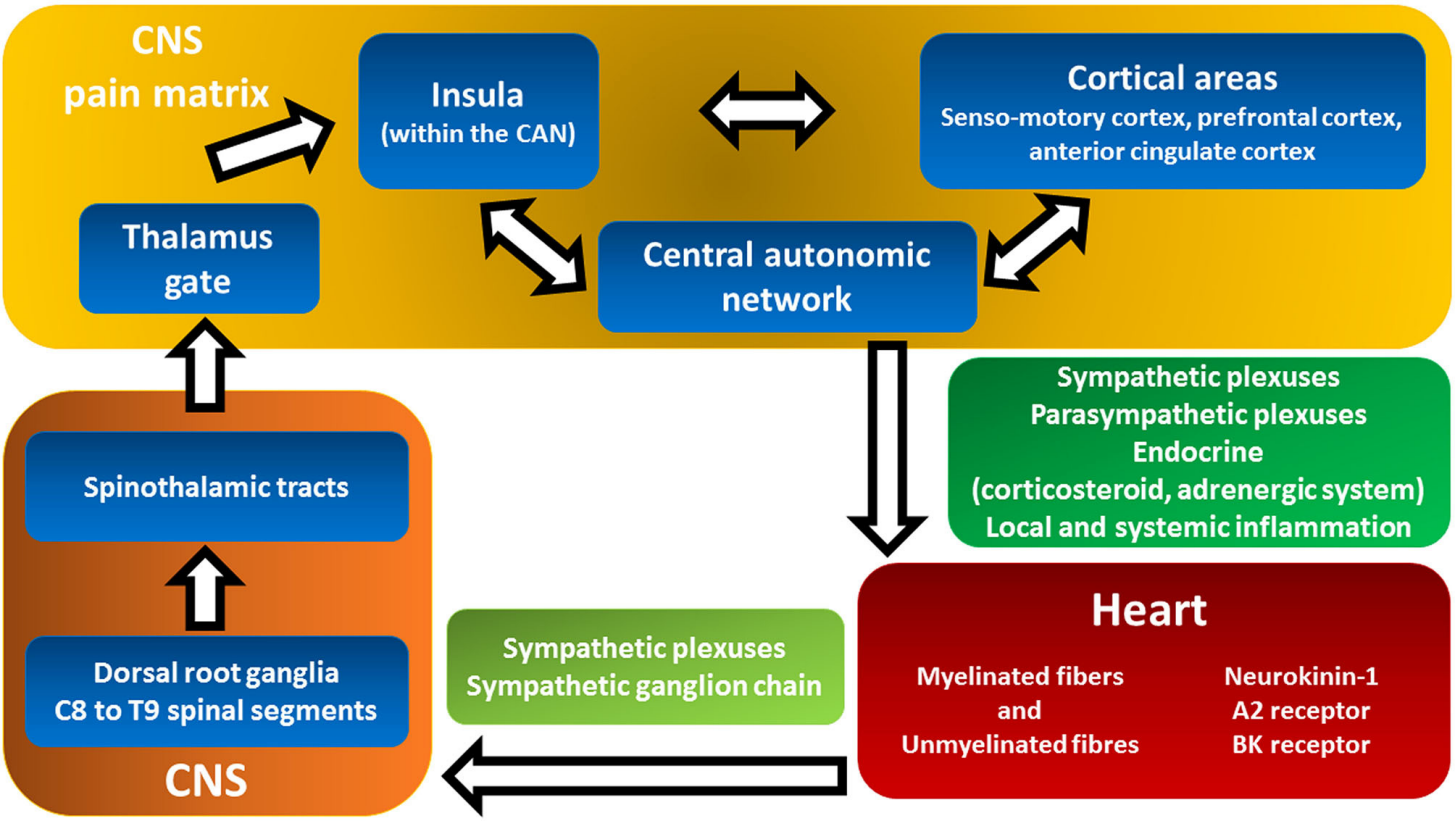
Cardiac pain transmission and modulation pathways from the heart to pain matrix in the central nervous system. This figure shows the complex and multilevel genesis and processing of cardiac pain and the associated symptoms triggered by ischemia. Myocardial-visceral pain perception (red box) is transmitted to and modulated by the pain matrix (yellow box) in an extremely broad range of conscious pain sensations that convoy a large spectrum of subject's negative experiences: dyspnea, fatigue, dyspepsia, fear, and sense of imminent death (see text for details). Backwards, the central autonomic network in the CNS exerts both local and systemic effects on the inotropic and chronotropic activity of the heart as well as coronary macro- and microcirculation (green box, see Figure 2 for details). A2 receptors, adenosine 2 receptors; BK, bradykinin; CAN, central autonomic network; CNS, central nervous system.

The heterogeneity and somewhat refractoriness of the above-mentioned symptoms reflect the complex genesis and processing of cardiac pain and the associated symptoms (Figure 3). Sensory nerve endings in the myocardium are not specific but rather a mix of myelinated and unmyelinated fibers associated with both chemosensitive and mechanosensitive nerve endings, mostly responding to bradykinin and adenosine (Figure 3, red box) (58).

The vagus nerve appears to have a minor role in afferent pain transmission. There are extensive connections among cardiac sympathetic plexuses, sympathetic ganglion chains, and the spinal cord (Figure 3, light green box). The cell bodies of sympathetic afferent fibers reside in the dorsal root ganglia of the C8–T9 spinal segments (Figure 3, orange box) (59). The ascending fibers continue mostly within the spinothalamic tracts (Figure 3, orange box) (59).

Studies investigating the projections of cardiac pathways to the brain showed that the thalamus plays a central role in gating the afferent pain signals (Figure 3, yellow box, Thalamus gate) (59, 60). Differently, the insula and other frontal cortical centers are considered responsible for pain sensation (Figure 3, yellow box) (59, 60). There is compelling evidence that the insula monitors common visceral sensations (61). Moreover, the insula is involved in the integration of cortical areas (sensomotory cortex, prefrontal cortex, and anterior cingulate cortex) and the modulation of the autonomic responses of the central autonomic network (CAN) (Figure 3, yellow box) (61). Myocardial-visceral pain perception is transmitted and modulated by the so-called pain matrix (Figure 3, yellow box) in an extremely broad range of conscious pain sensations (62). The system is even more complex since afferent neural cross talk occurs at both peripheral and central levels, thus expanding the possibilities of modulation and integration of stimuli that result in a large assortment of symptoms (59, 63).

#### Central Nervous System Functional and Structural Assessment

Multiple features of the role of the central nervous system and the autonomic system have been explored in subjects with MVA. Autonomic cardiac control imbalance has been investigated in small studies. Non-invasive surrogates for cardiac autonomic function such as sympathetic skin response and heart rate variability assessed by frequency domain R-R interval variation have been reported to be impaired in subjects with MVA (64, 65). Moreover, Lee et al. have evaluated the temporal correlation between dynamic changes in cardiac autonomic control, assessed by HRV, and myocardial ischemia on 24-h ECG monitoring in 34 consecutive drug-free patients with MVA (64). These results argue on the potential autonomic disarrangement preceding episodes of ischemia detected by ECG monitoring. However, evidence is scant and non-conclusive, since other authors have not found signs of autonomic dysfunction despite altered coronary vascular resistance indicating microvascular dysfunction (66).

Since 1980s, there have been studies suggesting an abnormal increase in the perception of cardiac pain in patients with MVA, even during typically painless intracardiac stimuli (67–69). This evidence supports the hypothesis that a reduced cardiac pain threshold may contribute to the clinical manifestations of MVA. However, it has not been clarified whether the cause of this enhanced perception is peripheral, within the cardiac nervous system, or central, as well as whether this is a specific cardiac feature or a component of a generalized pain disorder.

Using a randomized, double-blind design, Pasceri et al. found that enhanced cardiac pain perception was confined to the ventricular myocardium (70). Consistently, severely impaired cardiac MIBG uptake has been shown in MVA. Functional abnormalities in afferent cardiac adrenergic nerve fibers support the hypothesis of a primary role of the cardiac nervous system in the pathogenesis of MVA (71). However, our group assessed general sensitivity to pain in a small group of subjects by forearm ischemic pain test (FIP) without any significant difference in results among MVA, AMI, and Takotsubo cardiomyopathy groups (40).

Conversely, the study by Frøbert et al. found that the central nervous system response to multiple visceral and somatosensory nociceptive stimuli is altered in patients with MVA, thus advocating the hypothesis of a generalized pain disorder (66). Moreover, literature further supports the role of CNS abnormalities in the pain modulation and transmission. Rosen et al. found transient increase in perfusion in specific cortical afferent centers by positron emission tomography (PET) in conjunction with ST-segment depression during a dobutamine stress-test (72). Since increased perfusion means an increased neural activity, this study suggests that cortical pain modulation may have a prevalent role in pain modulation in MVA. The work by Valeriani et al. implemented cortical laser evoked potentials (LEPs) to show that central modulation of painful stimuli was characterized by inadequate habituation in 13 patients with refractory angina and MVA as compared to 10 patients with refractory angina due to obstructive CAD (73). This evidence suggests that the role of CNS in pain modulation and transmission is independent from the burden of symptoms. On the contrary, the CNS may contribute to enhance these clinical manifestations.

SPECT cerebral nuclear perfusion imaging showed a high incidence of hypoperfusion lesions in the cortical parietal lobes coincident with myocardial defects (74).

Most likely, the complex process of pain transmission and modulation may be abnormal at multiple levels. Presumably, different components of the pain matrix may have variable relative contributions in different subjects and within the same subject at different times (Figure 3).

Notably, pain processing may not represent the sole abnormality in the CNS functional network. Convincing evidence exists that psychosocial stress perturbs the CAN, which in turn modulates autonomic response to external stressors (24). Recently, our group implemented resting-state brain functional MRI to investigate the CAN in patients with MVA. The exploratory screening of the whole functional organization of the CAN did not show any specific functional organization under resting conditions. We hypothesized that triggered functional CAN dysfunction rather than either structural or functional predisposing abnormalities within the CAN may contribute to the clinical manifestations of MVA (40).

### Treatment: Refractory Angina

In patients with chronic coronary syndrome (CCS) and obstructive CAD, current medical management and revascularization result in symptom control in about 80% of patients (8, 75). While optimal conventional medical therapy is available, a heterogeneous group of patients with refractory angina and angina-like symptoms still present with debilitated HRQoL in both the physical and mental health domains. Refractory angina is often present in subjects with MVA who often report atypical symptoms.

The management of refractory angina is particularly challenging with conventional pharmacological therapies. Therefore, a repertoire of unconventional pharmacological and non-pharmacological interventions has been investigated. These studies suggest that non-pharmacological psychoeducational interventions may improve quality of life and reduce health care demands to some extent, even without change in the ischemic threshold, presumably by modifying the complex pathways of pain transmission and modulation in the cortical and subcortical CNS (Table 1). However, as discussed later, the results of these pharmacological and non-pharmacological interventional trials have been limited by variable patient selection, the lack of functional study of coronary circulation, small sample size, and short follow-up (89) (Tables 1–**3**).

**Table 1:** Interventional studies investigating psychoeducational intervention in MVA.

**References**	**Trial type**	**Inclusion criteria**	***N***.	**F%**	**Age**	**Intervention**	**Fu**	**Assessment methods**	**Endpoints**
Asbury et al. (76)	Randomized non-blinded	Chest pain Positive EST Normal CA	49	100%	61.8 ± 8	Support group	12 month	HAQ HADS SF-36 YABS ESSI	Higher social support Improved QoL
Asbury et al. (77)	Randomized non-blinded	Chest pain Positive EST Normal CA	64	100%	57.3 ± 8.6	Psychological intervention during cardiac rehabilitation	8 weeks	HADS HAQ SF-36	Improves exercise tolerance, Improved QoL Less symptom severity
Tyni-Lenne et al. (78)	Single-blind, randomized controlled	Chest pain Positive EST Normal CA	24	100%	41–65	Physical training with relaxation therapy and physical therapy	8 weeks	VO_2_max SCI SIP	Physical training improve exercise capacity and QoL Relaxation improve QoL
Cunningham et al. (79)	Non-randomized, non-blinded	Chest pain Positive EST Normal CA	9	100%	56 (48–66)	Transcendental meditation (3-month course)	3 weeks	ST-segment depression Bruce protocol; Exercise duration before symptoms; Chest pain diary	Improved exercise tolerance Less angina episodes
Mao et al. (80)	Non-randomized, non-blinded	Chest pain Positive EST Normal CA	51	78%	51 ± 6	Liqi Kuanxiong Huoxue method of TCM	2 weeks	METs tredmill test	Higher exercise capacity
Bi et al. (81)	Non-randomized, non-blinded	Chest pain Positive EST Normal CA	51	58%	18–74	Qi-regulating, chest-relaxing and blood-activating therapy of TCM	8 weeks	Angina diary METs hs-CRP	Reduction angina frequency
Asbury et al. (82)	Randomized, non-blinded	Chest pain Positive EST Normal CA	53	100%	57.1 ± 8	Autogenic training	8 weeks	Chest pain diary HADS STAI CAQ QLI	Improves symptom frequency
Potts et al. (83)	Randomized, non-blinded	Chest pain Positive EST Normal CA	60	63%	52.8 ± 8	Cognitive behavioral therapy	6 months	HADS NHS SIP NHP Chest pain diary	Improve anxiety, depression scores, disability rating and exercise tolerance Unchanged positive EST

*CA, coronary angiography; F%, female percentage; FU, follow-up period; hs-CRP, high sensitivity C-reactive protein; METs, metabolic equivalent of oxygen; n., number; QoL, quality of life; TCM, traditional Chinese medicine*.

*Clinical scales: 36-Item Short Form Health SurveySF-36; CAQ, Cardiac Anxiety Questionnaire; ESSI, ENRICHD Social Support Instrument; EST, ECG stress test; HAQ, Health Anxiety Questionnaire; HAQ, Health Anxiety Questionnaire; HADS, Hospital Anxiety and Depression Scale; NHS, Nijmegen Hyperventilation Scale; NHP, Nottingham Health Profile; QLI, Powers Quality of Life Index; SIP, Sickness Impact Profile; STAI, State-Trait Anxiety Inventory; SCI, Stress and Crisis Inventory; YABS, York Angina Beliefs scale*.

### Treatment: Psychoeducational Interventions

#### Interventions During Cardiac Rehabilitation

In the last decade, cardiac rehabilitation has gained a role of primary importance in coronary heart disease and chronic heart failure management.

Cardiovascular rehabilitation has been shown to counteract deconditioning and reduce angina in MVA (95, 96). Likewise, a randomized, non-blinded study (77) and a single-blind, randomized trial (78) in middle-aged women diagnosed with MVA suggest that psychological intervention and relaxation therapy during cardiac rehabilitation improve short-term exercise tolerance, HRQoL, and symptom severity as assessed by largely validated questionnaires. Local experiences have been conglomerated into specific programs of pragmatic rehabilitation for refractory angina, namely programs constituted by medical, pharmacological, and psychoeducational interventions to manage refractory symptoms (97, 98).

Similarly, non-blinded, randomized evidence in females reinforced the role of support groups in MVA management. Support groups may enhance social participation, problem discussion, and may improve different items of the SF-36 questionnaire at 12 months (76).

Altogether, these data suggest that non-pharmacological interventions consisting of social and psychological support may reduce health care demands and improve quality of life in MVA at least at short- and mid-term follow-up (Table 1).

#### Cognitive-Behavioral Therapy

Cognitive-behavioral therapy (CBT) consists of a program of education, relaxation exercises, coping skills training, and stress management interventions (99). CBT is an evidence-based treatment for a few psychiatric disorders, including depression, generalized anxiety disorder, and obsessive-compulsive disorder that aims at reducing symptoms and enhancing functioning. CBT for angina consists of counseling and education about cardiovascular disease and angina, stress management techniques, and relaxation techniques. In this setting, CBT aims at regaining the activities avoided because of chest pain and concomitant negative experiences. In women with suspected MVA, an 8-week CBT program reduced patients' anxiety, disability, and increased exercise tolerance despite an unchanged prevalence of ECG-positive exercise stress-test at 6 months (83) (Table 1). Obviously, these are preliminary results. Actually, CBT is known to be more effective in highly motivated individuals and problem-solving individuals, and it depends on the practice within and outside of the therapy setting.

#### Transcendental Meditation and Traditional Chinese Medicine

The practice of meditation dates back a few thousand years and is mostly associated with Eastern philosophies and religions, although it is also present in the Middle Eastern and Western religions.

Meditation is increasingly practiced, and modern approaches concentrate principally on focused attention and mindfulness (15).

Cunningham et al. showed that transcendental meditation may improve exercise capacity without changes in ST-segment depression or reduction of angina frequency in postmenopausal women with MVA (79).

Similarly, traditional Chinese medicine (TCM) is a millennial traditional that views the human body as a complex dynamical system and focuses on the balance of the human body within itself and its environment. Such concepts require investigations that go beyond conventional reductionism. Therefore, prove effectiveness of TCM *via* scientific trials is still a challenge. Two small non-randomized studies used the Qi-regulating, chest-relaxing, and blood-activating therapies of the TCM integrated with Western Medicine for the treatment of MVA, suggesting a potential benefit on symptoms, exercise capacity, and systemic inflammation assessed by sensitive C-reactive protein (CRP) (81).

Although the methodological limitation of these small pilot studies must be recognized, these results argue for the potential usefulness of integrated psycho-physiological interventions that involve cortical, autonomic, neuroendocrine, and cardiovascular systems (Table 2). Independently from the current level of evidence, it is estimated that 8% of US adults practice some form of meditation (15). Therefore, these techniques should be taken into account in future research.

**Table 2:** Interventional studies investigating intervention on the central nervous system in MVA.

**References**	**Trial type**	**Inclusion criteria**	***N***	**F%**	**Age**	**Intervention**	**Fu**	**Assessment methods**	**Endpoint**
Cox (84)	Randomized, double-blind, cross-over	Chest pain Positive EST Normal CA	18	100%	53 (35–72)	Imipramine 50 mg vs. placebo	5 weeks	NHP Angina diary	Reduced angina episode No improvements in QoL
Cannon et al. (85)	Randomized, double-blind	Chest pain Positive EST or SPECT Normal CA	60	66%	50 (29–72)	Imipramine 50 mg vs. Clonidine 0.1 mg vs. placebo	3 weeks	Angina diary	Reduced angina episode
Jessurun et al. (86)	Non-randomized, unblinded	Chest pain Positive PET Normal CA	8		55 ± 7	TENS	4 weeks	Angina diary PET flow/perfusion imaging (dipyridamole stress test)	Reduction of angina episodes Reduction weekly nitroglycerin Increased PET perfusion reserve ratio between rest and dipyridamole
de Vries et al. (87)	Non-randomized, unblinded	Chest pain Positive EST or SPECT Normal CA	36	62.5%	56.7 ± 8	TENS, Cross-over to SCS when TENS not tolerated	5 years	SAQ Anti-anginal medications	Improved SAQ domain “disease perception,” “physical limitation,” “anginal frequency” Reduced nitroglycerin consumption
Rosano et al. (88)	Non-randomized Parrel group (MVA, CAD, controls)	Chest pain Positive EST Normal CA	25	42%	48 ± 8	Neuropeptide Y coronary infusion	0	CA	Prolonged constrictor response to NPY in MVA

*CA, coronary angiography; EST, ECG-stress test; F%, female percentage; FU, follow-up period; MVA, primary microvascular angina; n., number; NHP, Nottingham Health Profile; NPY, neuropeptide Y; PET, positron emission tomography; QoL, quality of life; SAQ, Seattle Angina Questionnaire; SCS, spinal cord stimulation; SPECT, single photon emission tomography; TENS, trans-cutaneous electrical nerve stimulation*.

### Treatment: Neuromodulation

#### Tricyclic Antidepressants

Tricyclic antidepressants (TA) have a balanced reuptake inhibition of the serotonin and noradrenaline neurotransmitter activities that results in an analgesic effect (100). Two short-term blinded, placebo-controlled trial studies showed a potential benefit of imipramine on angina episodes in patients with MVA (Table 2). The study by Cannon et al. (85) included subjects with angina chest pain and either positive or negative functional imaging (SPECT). Imipramine use resulted in a 52% decrease in chest pain episodes at 3 weeks, irrespective of the cause of the chest pain. However, no information on HRQoL was provided. Cox et al. showed that imipramine treatment decreased angina frequency in MVA at 5 weeks but reported a failure to improve HRQoL (101). The failure to demonstrate an associated improvement in quality of life was probably due to the non-negligible high incidence of anticholinergic side effects (dry mouth, dizziness, nausea, and constipation) typical of TA. Taking into account these common adverse reactions, the numerous interactions, and the potential for cardiovascular toxicity of TA, different pharmacological treatments implemented in neuropathic chronic pain syndrome, such as Duloxetine might be more tolerable alternatives to be investigated.

#### Spinal Cord Stimulation

Spinal cord stimulation (SCS) is a neuromodulation technique used in various chronic pain syndromes. SCS for modulation of cardiac pain requires surgical positioning of a multipolar electrode in the epidural space between the C7 and T4 vertebrae where the myocardial afferent sympathetic fibers synapse in the dorsal horns. During SCS, the electrode is connected to a programmable pulse generator that allows the subject to self-administer a customized stimulation, which usually requires fixed daily stimulations and stimulations on demand whenever angina occurs (102). SCS acts on the dorsal-horn of the spinal cord, and it is thought to antagonize the effect of the descending inhibitory pathways that are known to favor the transmission of nociceptive stimuli (103–105). In addition, SCS has been hypothesized to mediate a sympatholytic effect additive to the analgesic effect (103, 106). However, the studies by Lanza et al. (94) and Spinelli et al. (107) suggested that SCS effects principally rely on the modulation and transmission of peripheral cardiac pain stimuli rather than being mediated by modulation of cardiac adrenergic nerve activity in MVA.

Evidence from small observational studies showed that SCS may be an effective and safe treatment for refractory angina in MVA both at short- and long-term follow-up (108). SCS is the unconventional, non-pharmacological intervention with the widest evidence in MVA (Table 3). Nonetheless, most interventional studies were performed in the 2000s, and they are non-randomized and non-blinded. Short follow-up characterized these studies, with the exception of the non-randomized work by Sgueglia et al. (93) that followed carefully selected patients with MVA and refractory angina for a mean of 8.5 years, showing that SCS may lower angina frequency, angina duration, nitrate use, and it may improve Seattle angina questionnaire (SAQ) functional status and quality of life. Importantly, unlike previously discussed interventions, studies investigating SCS had a prevalence of female subjects similar to that of the large prospective registries (Table 3). Interestingly, Lanza et al. (92) showed that SCS reduced the number, duration, and severity of angina episodes, the episodes of ST-segment depression on 24-h Holter monitoring, as well as increased time to angina and time to ST-segment depression dobutamine ECG stress test (EST) at 6 weeks in patients diagnosed with MVA and refractory angina. Despite a short follow-up, the study had a randomized, single-blinded crossover design and required either a positive EST or SPECT as inclusion criteria, thus providing more reliable results. Similarly, the older study by Eliasson et al. (90) was characterized by refined selection criteria, including positive 12-lead-EST or SPECT or stress echocardiography and coronary vasomotor tests excluding vasospasm. The study included only 12 patients in a non-randomized, non-blinded design, but SCS provided relief of symptoms and improves exercise tolerance at 8 months in this carefully selected MVA sample.

**Table 3:** Interventional studies investigating spinal cord stimulation in MVA.

**References**	**Trial type**	**Inclusion criteria**	***N***	**F%**	**Age**	**Follow-up**	**Assessment methods**	**Endpoint**
Eliasson et al. (90)	Non-randomized Non-blinded	Chest pain Positive EST or SPECT or stress TTE Normal CA Coronary vasomotor test	12	75%	61 ± 6	8 months	EST duration EST ST-changes	SCS relief symptoms and improve exercise tolerance
Lanza et al. (91)	Non-randomized Non-blinded	Refractory angina Positive EST Normal CA	7	43%	59.3 ± 11	2–17 months	VAS Treadmill test Angina diary	Reduced number, duration, and severity of angina episodes Increased exercise tolerance
Lanza et al. (92)	Randomized Single blinded Cross-over	Chest pain Positive EST or SPECT Normal CA	10	70%	58.6 ± 5.7	6 weeks	SAQ VAS Holter monitoring D-EST	Reduced number, duration, and severity of angina episodes Reduced episodes of ST-segment depression on HM Increased time to angina DST Increased time to ST-segment depression DST
Sgueglia et al. (93)	Non-randomized Non-blinded	Refractory angina Chest pain Positive EST Normal CA Excluded CS SHD	28	73%	60.9 ± 8	8.5 years	SAQ VAS	Lower angina frequency, duration Lower nitrate use Improved SAQ functional status Improved VAS QoL
Sestito et al. (94)	Randomized Cross-over	Chest pain Positive EST Normal CA	16	75%	61.6 ± 7	0	Laser evoked potentials	Restore habituation to peripheral pain stimuli

*CA, coronary angiography; D-EST, dobutamine ECG stress test; EST, ECG stress test; F%, female percentage; FU, follow-up period; MVA, primary microvascular angina; n., number; stress ETT, stress transthoracic echocardiography; SAQ, Seattle Angina Questionnaire; SHD, structural heart disease; SPECT, single photon emission tomography; VAS, EuroQoL Visual Analog Scale*.

#### Transcutaneous Electrical Nerve Stimulation

Transcutaneous electrical nerve stimulation (TENS) is a non-invasive, self-administered technique to relieve pain. TENS consists of pulsed electrical impulses delivered across the intact skin to selectively activate large-diameter non-noxious afferent fibers to antagonize nociceptive afferent transmission. Pain relief with TENS is rapid in onset and offset. Clinical experience suggests potential beneficial effect for acute pain management, although data are conflicting and evidence in MVA is limited. Two small non-randomized, non-blinded studies including MVA provided preliminary data on both short- and long-term beneficial effects of TENS in reducing physical limitation, angina frequency, and possibly the perfusion reserve assessed by dipyridamole positron emission tomography (86, 87) (Table 2). Notably, the sole study providing long-term follow-up (mean 5 years) was characterized by a high rate of crossover from TENS to SCS (about 50%) and a significant drop-out rate (87).

#### Cardiac Sympathectomy

The left stellate (cardiothoracic) ganglion is an accessible station of convergence for afferent cardiac pain. Temporary and permanent sympathectomy by stellate ganglion blockade and resection, respectively, have been attempted for channelopaties and structural arrhythmias management. Anecdotal experience in patients with refractory angina has been reported, but no suitable evidence exists for the management of MVA.

## Limitations and Gaps in Evidence

Most of the studies included in this review are characterized by low sample size, present cross-sectional design, and rarely a crossover design. Therefore, a potential for bias exists. This challenges the attempt to establish causal associations.

Most randomized interventional trials have short follow-up, and the few trials characterized by mid- and long-term follow-up are non-randomized.

Most studies required a positive EST to detect ischemia. However, the EST is known to yield a lower diagnostic accuracy compared with diagnostic imaging tests and large studies found that a positive exercise stress test was neither sensitive nor specific for MVA (22, 109). Therefore, there could have been an overdiagnosis of MVA among subjects participating in both the observational and interventional studies. Moreover, INOCA comprises different endotypes (3, 4). Epicardial vasospastic angina (VSA) and MVA may manifest with indistinguishable clinical characteristics. Most of the studies present in the review have been published in the 1990s and 2000s, and inclusion criteria did not require functional assessment of coronary artery anatomy with few exceptions. The importance of functional assessment in INOCA and MVA has been recently stressed in international consensuses (3, 4). Therefore, VSA and MVA could have been misdiagnosed and most patients included in these studies could have fit the current definition of possible MVA (3), thus hampering inference regarding the role of psychosocial factors and the central nervous system in MVA.

Finally, medical community is still slowly gaining awareness that MVA without CAD represents a separate condition when compared to MVA with non-obstructive CAD, with different prognostic and potentially therapeutic implications (110). The latter represents the largest cohort of patients with coronary microvascular dysfunction, and it cannot be clearly distinguished within the cohort of patients with MVA in the studies included in this study.

## Conclusion

There is evidence suggesting potential interactions between clinical manifestations of MVA and non-cardiac conditions such as abnormal function of the CAN, abnormal pain modulation pathways, and psychological, mental, and social conditions. A few non-pharmacological techniques targeting these psychosocial conditions and modulating the CNS pathways have been proposed to improve symptoms and quality of life. Most of these unconventional approaches have shown encouraging results. However, the results of the available observational and interventional trials have been limited by variable patient selection, the lack of a standardized diagnosis, inadequate small sample size, and insufficient follow-up.

Although there is a slow increase in awareness of the importance of microvascular dysfunction and MVA by the cardiovascular community, therapeutic success remains frustratingly low for both patients and physicians. Altogether, those considerations should promote basic and clinical research in this relevant cardiovascular field. Standardization of definitions, clear pathophysiological-directed inclusion criteria, crossover design, adequate sample size, and mid-term follow-up through multicenter randomized trials are mandatory for future research in this field.

## Author Contributions

MC contributed substantially to the conception and design of the work, the acquisition, analysis and interpretation of data, to the draft and revision of the work, and provided approval for the publication of the content. GH, MMC, and AY contributed substantially to the acquisition, analysis and interpretation of data, to the draft and revision of the work, and provided approval for the publication of the content. IS and CG contributed substantially to the analysis of data, to revision of the work, and provided approval for publication of the content. AG contributed substantially to the conception and design of the work, the interpretation of data, to the draft and revision of the work, and provided approval for publication of the content. All authors contributed to the article and approved the submitted version.

## Conflict of Interest

The authors declare that the research was conducted in the absence of any commercial or financial relationships that could be construed as a potential conflict of interest.

## Publisher's Note

All claims expressed in this article are solely those of the authors and do not necessarily represent those of their affiliated organizations, or those of the publisher, the editors and the reviewers. Any product that may be evaluated in this article, or claim that may be made by its manufacturer, is not guaranteed or endorsed by the publisher.
