# Supplementary material for: The Central Nervous System and Psychosocial Factors in Primary Microvascular Angina
Source: Front Cardiovasc Med. 2022 May 13;9:896042. doi: 10.3389/fcvm.2022.896042 (PMC9136057; doi:10.3389/fcvm.2022.896042)
Supplement: Supplementary file 1 [file Data_Sheet_1.DOCX]

**Supplemental methods**

**Search terms and date**

The list of search terms in provided below. Cardiac syndrome X, primary microvascular angina and ischemia with non-obstructive coronary arteries (INOCA) were all included in the research as population of interest. Medline, Scopus, Embase, Web of science databases were consulted from December 30^th^ 2021 to January 6^th^ 2022. Moreover, we examined reference lists cited in study reports.

Cardiac syndrome X OR Primary microvascular angina OR Ischemia with no obstructive coronary arteries

AND Diagnostic and Statistical Manual of Mental Disorders

AND anxiety

AND depression

AND schizophrenia

AND personality disorders

AND somatoform disorders

AND type D personality disorder

AND fear

AND anger

AND joy

AND substance disorders

AND psychotic disorders

AND psychosocial stress

AND socioeconomic determinants

AND socioeconomic status

AND work stress

AND educational level

AND psychosocial factors

AND social support

AND loneliness

AND critical life events

AND vital exhaustion

AND insomnia

AND pain modulation

AND pain transmission

AND pain perception

AND central nervous system

AND spinal cord

AND stellate ganglion OR ganglion

AND cerebral magnetic resonance imaging

AND psychotherapy

AND behavioral therapy

AND cognitive behavioral therapy

AND psychotherapy

AND psychoeducational intervention during cardiac rehabilitation

AND Yoga

AND Autogenic training

AND Hyperventilation control

AND hypnotherapy

AND counselling

AND traditional Chinese medicine

AND stellate ganglion ablation

AND spinal cord modulation

**Eligibility criteria and information source**

All sub-types of retrospective and prospective observational studies on humans were considered eligible for inclusion. Studies with both cross-sectional and longitudinal design were included. We include full-text reports without year of dissemination criteria. No language criteria was implemented during search to include reports published in two languages, but only reports including a full text English version were considered eligible for inclusion.

Moreover, we considered eligible randomized and non-randomized trials recruiting patients diagnosed with MVA and investigating the effect of the following intervention on anginal symptoms and quality of life without a minimum follow-up restriction: stellate ganglion ablation, spinal cord modulation (either spinal cord stimulation or transcutaneous electrical nerve stimulation), cognitive behavioral therapy, psychotherapy, hypnotherapy, yoga, autogenic training, hyperventilation control, counselling, traditional Chinese medicine, psychoeducational intervention during cardiac rehabilitation. Clinical endpoints of the studies should have included at least either angina frequency, or nitrate consumption, or quality of life assessment, or anxiety scales, or depression scales, or exercise capacity, or disability rating, or change in prevalence of positive ECG-stress test (EST), or change in time to positive EST.

**Data selection and data collection process.**

Two reviewers selected independently each record (title/abstract) and each report retrieved, during screening and exclusions verification. Disagreements between screeners were resolved by consensus with a third author. One out of three reviewer independently collected data from report and two reviewers independently checked each report and disagreements were resolved by intervention of the third reviewer.

**Data items**

The results of the studies were divided according to the domains assessed as follows:

- Symptoms (chest pain)
- Quality of life assessment
- Chronic psychological stress
- Psychiatric co-morbidities
- Social and educational factors
- Central nervous system functional and structural assessment

Moreover, the results of the studies were divided according to design of the study as follows:

- Observational
- Interventional
